# Supporting open science practices at multidisciplinary scale

**DOI:** 10.1371/journal.pone.0355163

**Published:** 2026-08-07

**Authors:** Devin Soper, Lauren Cadwallader, Iain Hrynaszkiewicz

**Affiliations:** 1 Public Library of Science, San Francisco, California, United States of America; 2 Public Library of Science, Cambridge, United Kingdom; PLOS: Public Library of Science, UNITED KINGDOM OF GREAT BRITAIN AND NORTHERN IRELAND

*PLOS One* is widely recognized as the world’s first megajournal, but its pioneering role in advancing open science is perhaps less well known. From the outset, *PLOS One*’s multidisciplinary scope provided a unique opportunity to design open science policies and innovations that could benefit researchers across fields, institutions, and regions. Over the years, the journal has implemented changes that have significantly broadened access to research outputs beyond the article such as research data, preprints, protocols, peer reviews, and study registrations – and promoted inclusion in research. Here, we reflect on this legacy of openness and innovation, drawing on evidence from the PLOS Open Science Indicators (OSI) dataset [1] to consider how open science practices among *PLOS One*’s broad multidisciplinary author community have evolved at the journal over time.

## 2014: Data Availability Policy

The implementation of the PLOS Data Availability Policy in 2014 formalized in all publications its requirement for public data sharing and introduced new mandatory data availability statements [2]. The adoption of the policy at *PLOS One* is notable because it extended the new requirements to so many different disciplines at once. This was the first time that a mandatory data availability policy had been implemented at such a broad, multidisciplinary scale.

It is perhaps fitting, then, that the PLOS Data Availability Policy foregrounds author choice: instead of mandating deposit in data repositories, the policy allows authors to share data as part of their supplementary information files, removing a key point of friction especially for authors in fields that lack disciplinary data repositories or experience with data sharing. Even though PLOS would prefer that all research data be openly shared in repositories and wants to support and encourage authors in moving toward that aspirational vision, we also believe that such a transition needs to happen equitably and meet researchers in different fields where they are, accommodating diverse needs and experience levels with respect to practicing data sharing. Accordingly, since the policy was adopted, the work that we have done to improve data sharing practices has been based on providing new incentives for good practices, as with the implementation of our Accessible Data icon in 2022 [3].

PLOS OSIs show that there has been steady growth in the proportion of papers that share data in repositories (from 18.4% in 2018 to 28.5% in 2025) and a declining proportion that share data as supplementary information files (from 63.9% in 2018 to 51.2% in 2025), which in turn suggests that *PLOS One* authors have grown more comfortable with best practices for data sharing over time.

**Fig 1:**
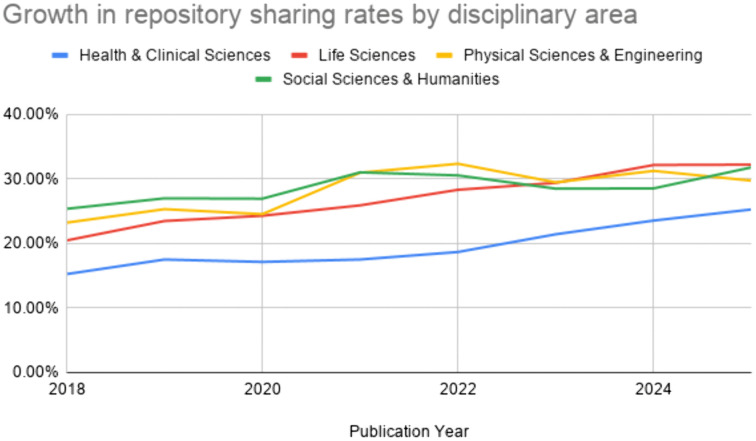
Growth in data shared in repositories by *PLOS One* authors relative to other methods of data sharing by broad disciplinary area and year.

Reassuringly, this trend holds across broad disciplinary areas: with the slight exception of the physical sciences, for which sharing rates have declined marginally since 2021, more and more authors are depositing their data in repositories in alignment with best practices across the life sciences, health and clinical sciences, and social sciences and humanities (Fig 1). Changes in research practice and culture are rarely adopted quickly, but the changes observed since *PLOS One*’s Data Availability Policy show positive change happening at multidisciplinary scale.

## 2016: Preprint Server Integrations

PLOS has long supported preprint sharing due to their potential to accelerate the dissemination of new research, improve the visibility and impact of the papers we publish, democratize the peer-review process, and increase the transparency of the scholarly record. *PLOS One* formalized its support for preprints in 2016, when the journal began accepting submissions via direct transfer from bioRxiv [4]. Since then, the journal has implemented a range of additional solutions to encourage adoption of preprint sharing among its authors, including a new facilitated preprint posting service in 2018 that provided authors with the option to have their submission posted to bioRxiv on their behalf [5], a direct transfer service from medRxiv in 2019 [6], and a facilitated posting service to medRxiv in 2022 [7].

The primary purpose of each of these solutions is to make it easier for our authors to share their manuscripts as preprints, whether before or after submission to *PLOS One*, with the goal of improving the adoption of preprint sharing at the journal over time.

**Fig 2:**
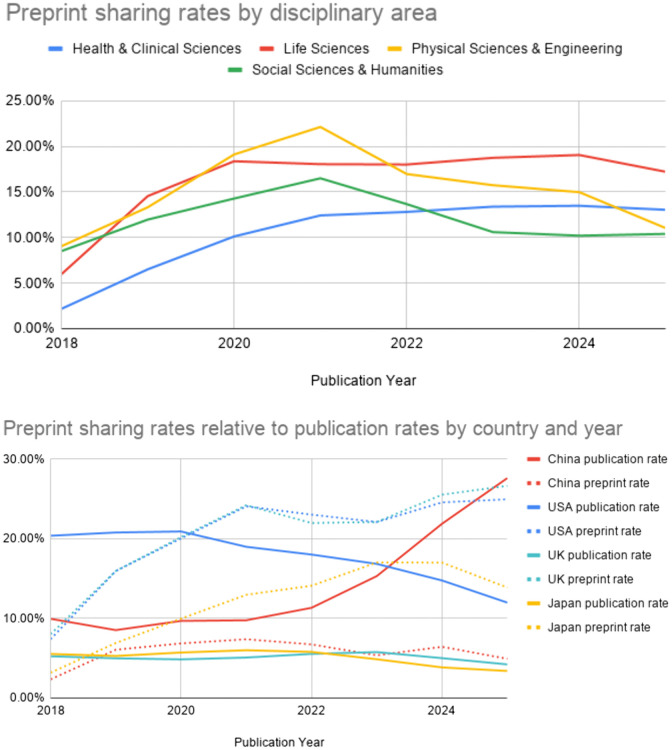
*PLOS One* preprint sharing rates by year and disciplinary area juxtaposed against preprint sharing and publication rates by corresponding author country and year.

Preprint sharing rates at *PLOS One* improved significantly across disciplinary areas from 2018 through 2021 (Fig 2). Much of this improvement occurred before the COVID-19 pandemic, which heralded increased scientific and public engagement with preprints [8], suggesting that the preprint sharing solutions implemented at *PLOS One* did initially help to drive adoption. Since 2021, however, preprint sharing rates have been flat or declining at the journal across all disciplinary areas, with rates among life sciences and health sciences publications remaining mostly consistent, and with rates among physical sciences and social sciences and humanities publications declining close to 2018 levels.

Although we cannot say exactly why the growth in preprint sharing rates through 2021 has since slowed or declined, changes in the population geography of *PLOS One* authors over time appear to tell part of the story (Fig 2). Specifically, publications by corresponding authors based in China have grown substantially, and preprint sharing rates among these authors are lower compared to those of authors based in the next four largest corresponding author countries by *PLOS One* publication count. While there are likely other contributing factors, we are encouraged that a significant proportion of *PLOS One* authors continue to share their manuscripts as preprints. We will continue to explore ways to support authors in this important open science practice, including expanding facilitated preprint posting services to additional servers such as EarthArXiv.

## 2019−20: New Article Types

*PLOS One* has also introduced new article types designed to promote rigour and encourage the dissemination of research outputs beyond the article. The first of these, Registered Reports, was launched in 2020 to combat publication bias and increase transparency and reproducibility through study registration (or preregistration), the practice of publicly sharing study design protocols prior to conducting research studies and writing up consequent findings. *PLOS One*’s Registered Reports article type includes two stages of publication, starting with submission of a Registered Report protocol that is submitted and reviewed before the authors begin their investigations, and ending with a Registered Report Research Article that is submitted after the authors have concluded their investigations and written up their findings. To date, *PLOS One* has published 62 registered report articles. In 2021 we launched another two article types, Study Protocols and Lab Protocols, to enable researchers to receive greater recognition for research methods. Lab Protocols were launched via a first-of-its-kind partnership with protocols.io. These articles also consist of two interlinked components: a step-by-step lab protocol shared on the protocols.io platform, and a peer-reviewed article that provides additional context and discusses applications, limitations, and expected results. To date, *PLOS One* has published 2,726 Study Protocol articles and 259 Lab Protocol articles. These new publishing options have offered greater incentives for open science to a subset of the community, and the PLOS OSI data on prepregistration and protocol sharing rates (6.1% and 6.5% respectively) suggest there is more that could be done to support these research practices.

## Looking ahead

As we look ahead to the next decade of *PLOS One*’s efforts to advance open science, we see many opportunities both to enhance existing policies and solutions and to explore and validate new innovations. One priority will be to better understand barriers to data sharing in repositories and to design targeted interventions that improve outcomes of our Data Availability Policy while remaining sensitive to disciplinary and regional differences. New preprint server integrations also offer an opportunity to re-energize uptake of preprint sharing, particularly in communities where adoption has historically been lower. Building on the success of the PLOS Redefining Publishing project [9], a research and design initiative aimed at increasing recognition for contributions beyond the article such as data and code, we also intend to prioritize research and engagement with *PLOS One* authors and editors to better understand code sharing practices across the journal’s multidisciplinary community and to assess whether it is ready for more formalized expectations in this area – as have been established recently at other broad-scope PLOS journals [10,11]. Together, these efforts reflect values and principles that are central to *PLOS One*’s mission: advancing open science at scale and across disciplines in a way that respects diverse disciplinary needs and meets researchers where they are. By continuing to combine policy, infrastructure, and evidence-driven iteration, *PLOS One* is well positioned to support a more open, transparent, reproducible, and equitable research ecosystem in the years ahead.

## Supporting information

S1 File One 20^th^ – data in repo by disciplinary area. Data underlying Fig 1, showing growth in data shared in repositories by PLOS One authors relative to other methods of data sharing by broad disciplinary area and year.
(CSV)

S2 File One 20^th^ – preprint sharing rates. Data underlying Fig 2a, showing PLOS One preprint sharing rates by year and disciplinary area.
(CSV)

S3 File One 20^th^ preprint sharing rates vs pub rates. Data underlying Fig 2b, showing preprint sharing and publication rates by corresponding author country and year.
(XLSX)

S4 File Lab and Study Protocol Publications. Data underlying publication counts for PLOS One lab protocol and study protocol articles types as reported in paper.
(XLSX)

S5 File Registered report publications. Data underlying publication counts for PLOS One registered report article type as reported in paper.
(XLSX)
